# Quantification of the Service Life Extension and Environmental Benefit of Chloride Exposed Self-Healing Concrete

**DOI:** 10.3390/ma10010005

**Published:** 2016-12-23

**Authors:** Bjorn Van Belleghem, Philip Van den Heede, Kim Van Tittelboom, Nele De Belie

**Affiliations:** 1 Magnel Laboratory for Concrete Research, Department of Structural Engineering, Faculty of Engineering and Architecture, Ghent University, Tech Lane Ghent Science Park, Campus A, Technologiepark Zwijnaarde 904, B-9052 Ghent, Belgium; bjorn.vanbelleghem@ugent.be (B.V.B.); philip.vandenheede@ugent.be (P.V.d.H.); kim.vantittelboom@ugent.be (K.V.T.); 2 Strategic Initiative Materials (SIM vzw), Project ISHECO within the Program “SHE”, Tech Lane Ghent Science Park, Campus A, Technologiepark Zwijnaarde 935, B-9052 Ghent, Belgium

**Keywords:** concrete cracking, autonomous healing, encapsulated polyurethane, chloride diffusion, service life prediction, life cycle assessment (LCA)

## Abstract

Formation of cracks impairs the durability of concrete elements. Corrosion inducing substances, such as chlorides, can enter the matrix through these cracks and cause steel reinforcement corrosion and concrete degradation. Self-repair of concrete cracks is an innovative technique which has been studied extensively during the past decade and which may help to increase the sustainability of concrete. However, the experiments conducted until now did not allow for an assessment of the service life extension possible with self-healing concrete in comparison with traditional (cracked) concrete. In this research, a service life prediction of self-healing concrete was done based on input from chloride diffusion tests. Self-healing of cracks with encapsulated polyurethane precursor formed a partial barrier against immediate ingress of chlorides through the cracks. Application of self-healing concrete was able to reduce the chloride concentration in a cracked zone by 75% or more. As a result, service life of steel reinforced self-healing concrete slabs in marine environments could amount to 60–94 years as opposed to only seven years for ordinary (cracked) concrete. Subsequent life cycle assessment calculations indicated important environmental benefits (56%–75%) for the ten CML-IA (Center of Environmental Science of Leiden University–Impact Assessment) baseline impact indicators which are mainly induced by the achievable service life extension.

## 1. Introduction

As the impact of construction activities on the environment is becoming clearer, increasing attention is paid to sustainable design. As such, it is logical that the construction sector is encouraged to develop innovative construction materials and techniques that are more durable and environmentally friendly. Since concrete production contributes 5% of the annual anthropogenic global CO_2_ production, any new effort that could reduce the material’s environmental impact would have an enormous effect [1]. While concrete is already categorized as one of the five longest lasting building materials [2], its durability is impaired through the easy formation of cracks in the concrete matrix. Corrosion inducing substances can enter the matrix through these cracks and cause concrete degradation and steel reinforcement corrosion. Consequently, the presence of cracks lowers the durability of concrete structures. Self-repair of concrete cracks is an innovative technique which has been studied extensively during the past decade and which may help to increase the sustainability of concrete. Making use of this technique will trigger the activation of an embedded mechanism upon crack appearance resulting in immediate repair of the crack. This will result in an important service life extension for reinforced concrete structures, answering the needs of environmental sustainability. Moreover, self-repair of cracks will have economic benefits as costs will be saved because less manual repair interventions will be needed.

The inspiration to develop self-healing materials comes from biological systems, which do have the natural ability to heal after being wounded. In fact, also in the case of concrete, some help from nature is obtained as concrete in itself possesses natural self-healing properties, i.e., autogenous healing of cracks. Due to ongoing hydration of clinker minerals or carbonation of calcium hydroxide, cracks may heal after some time [3]. However, autogenous healing is limited to small cracks (crack width <50 µm [4,5]), is only effective when water is available and is difficult to control. Therefore, some researchers attempt to improve the autogenous crack healing mechanisms through restriction of the crack width making use of fibers [6,7,8,9] or shape memory alloys [10]. Other researchers improve autogenous crack healing through improvement of the water supply. This is obtained by the addition of super absorbent polymers [11,12] or encapsulated water reservoirs [13,14]. Alternative attempts to stimulate autogenous healing, focus on the addition of agents which are able to promote the deposition of crystals inside the crack [15,16,17].

Some research studies even go one step further and really modify concrete elements in order to obtain autonomous repair of cracks. This is obtained through the use of embedded capsules [18,19,20] or vascular systems [21,22,23,24] which are filled with polymer based or bacteria based healing agents. Upon crack appearance, the capsules or the vascular network break and release the healing agent from the inside of the matrix into the crack. When a bacterial healing agent is released, the bacteria will be activated inside the crack when coming into contact with water and nutrients. Due to this, calcium carbonate crystals will be precipitated by the bacteria inside the crack which will result in crack closure [25,26]. For polymer based healing agents the reaction depends on the type of agent. While one component agents mostly react when coming into contact with moisture inside the matrix [21,27], two component agents need to contact the second component to allow a polymerization reaction. The second component can be mixed into the matrix [28] or can be available in separate capsules [29]. After reaction of the healing agent the crack is sealed off against the ingress of aggressive substances.

From the aforementioned mechanisms, encapsulation of polyurethane by brittle glass capsules is a promising approach. By means of acoustic emission analysis the rupture of the capsules upon crack formation has been proven [30] and computed tomography was used to prove that the polyurethane healing agent was released from the capsules and flew into the crack [20]. The water permeability of cracked concrete can be reduced by a factor 10^2^ to 10^4^ if encapsulated polyurethane is provided [20]. By neutron radiography it was shown that also the water uptake capacity was decreased significantly [31,32]. Moreover, more than 50% of the original strength and stiffness could be regained after self-healing [20]. Autonomous crack healing with encapsulated polyurethane also has a beneficial influence on the resistance against chloride penetration [33,34]. In the work of Van Belleghem et al. [34] it was shown that a reduction in total chloride content of more than 70% was possible due to autonomous crack healing. However, the experiments conducted until now did not allow for an assessment of the service life extension possible with self-healing concrete in comparison with traditional (cracked) concrete. The main aim of this paper is to provide a quantification of the service life extension for marine concrete subjected to chloride-induced steel corrosion and to determine the resulting environmental benefits.

The evaluation of the environmental sustainability of a material should always be done in an objective way. Since the end of the 1960s, a step-by-step methodology has been gradually developed to achieve this goal [35]. Nowadays, it is known as the life cycle assessment (LCA) methodology which has been standardized in ISO 14040-14044. Thus far, it has been used for assessing the environmental impact of all kinds of products and services preferably from the cradle to the grave. By the turn of the millennium, it also drew the attention of the construction sector [36]. Within the concrete industry it has mainly been used to assess the influence of strength and structure dimensioning [37,38] and the influence of partial cement replacement by industrial by-products with cementitious properties on the environmental score of the concrete [39,40,41,42,43,44,45]. An important conclusion that could be drawn from those studies was that adequate life cycle assessment on any type of potentially sustainable concrete should always account for its strength and service life performance [43]. The environmental sustainability of self-healing concrete should be studied in the same way.

Natural chloride diffusion tests on uncracked concrete as well as accelerated chloride diffusion tests on (un)cracked and self-healing concrete with two different encapsulated PU-based healing agents were done to obtain the necessary experimental input to a probabilistic prediction model based on Fick’s second law of diffusion and to estimate the time to chloride-induced steel depassivation of the embedded reinforcing steel. Subsequently, life cycle assessment calculations were done in the commonly used and well-accepted SimaPro 8 software cf. ISO 14040-14044. The calculations were performed for steel reinforced concrete slabs made with ordinary (cracked) and the self-healing concrete and compared with each other.

This study is only a part of the ISHECO project (impact of self-healing engineered materials on steel corrosion of reinforced concrete). As described in the ISHECO project outline, the service life extension is to be assessed with probabilistic prediction models that are commonly used in engineering model codes but also by means of dedicated and more accurate numerical modeling approaches. This paper only focuses on the first approach. The second modeling approach and a subsequent fine-tuning with the first approach are still under investigation and will be published at a later stage.

## 2. Materials and Methods

### 2.1. Concrete Mixture

It has been proven that concrete where a certain amount of cement is replaced by fly ash has a higher resistance to chloride penetration, which is beneficial for the durability [39,46,47,48]. Therefore, a fly ash containing concrete mixture as described by Van den Heede [39] was used within this study. The mixture had a water-to-binder (W/B) ratio of 0.41 and a fly ash-to-binder (FA/B) ratio of 15%, according to the k-value concept [49]. The mixture was designed to be a representative reference mixture for concrete in exposure class XS2, i.e., submerged reinforced concrete susceptible to corrosion initiated by chlorides. Table 1 shows the mix proportions and properties of the concrete mixture.

### 2.2. Self-Healing Mechanism

Some of the concrete samples contained cylindrical borosilicate glass capsules (length: 35 mm, internal diameter: 3 ± 0.05 mm, wall thickness: 0.175 ± 0.03 mm) filled with a one component polyurethane-based healing agent. Filling of the capsules was done by sealing one end with a methyl methacrylate (MMA) based glue, subsequently injecting the polyurethane precursor into the capsules and finally sealing the other end with the MMA based glue. Upon the occurrence of a crack in the concrete matrix, the capsules easily break and release their content into the crack. Two different polyurethane (PU) precursors were used for this research. The first healing agent is a non-commercial PU which was developed in the framework of another project (SHEcon—self-healing concrete for structural and architectural applications). Its viscosity at 25 °C is 6700 mPas. It essentially consists of a polyether polyol and methylene diphenyl diisocyanate (MDI). For application in self-healing concrete, this PU precursor reacts with the moisture in the concrete matrix and hardens into a solid flexible foam inside the crack within 48 h. The polyurethane is denoted as PU_HV (High Viscosity). The second healing agent is a commercially available PU precursor with an approximate viscosity of 200 mPas at 25 °C. It is also a MDI and polyether polyol based prepolymer. In addition, it contains inert hydrophobic compounds that control the viscosity and rheological behavior. The viscosity of this second healing agent is more than 30 times less than the viscosity of the first one. Therefore, this product will be named PU_LV (Low Viscosity). For crack healing in concrete, this PU precursor flows into the crack and cures to a closed-cell polymer upon contact with moisture in the concrete matrix.

The viscosity of the healing agent is an important parameter for self-healing concrete. The viscosity should not be too high, in order to be able to flow out of the capsules and fill the crack. On the other hand, when the viscosity is too low it may leak out of the crack or be absorbed by the surrounding matrix [50]. According to Dry [51] the viscosity of the healing agent should be between 100 and 500 mPas. The commercial polyurethane precursor (PU_LV) has a viscosity in this range. However, the non-commercial product (PU_HV) with a very high viscosity was also used in the research, because good healing behavior was found with this polyurethane in previous research [32,52].

### 2.3. Curing, Sample Preparation, Cracking and Healing

For the natural chloride diffusion tests on uncracked concrete, a series of cylindrical concrete samples (height: 50 mm, diameter: 100 mm) were drilled from cubes (side: 150 mm) that were cured at 20 °C and 95% relative humidity until the age of 21 days and then partially coated with an epoxy coating on all sides except for the test surface to allow for a unidirectional chloride ingress during exposure.

**Figure 1:**
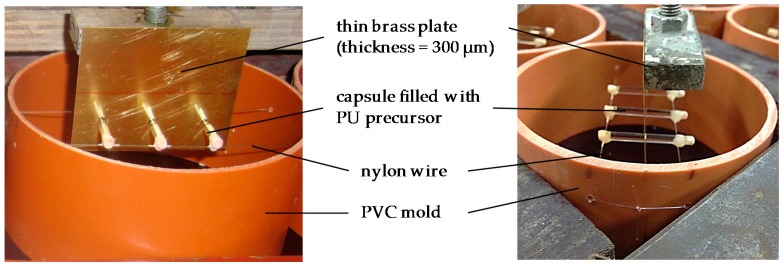
Preparation of self-healing concrete samples.

For the accelerated chloride diffusion tests, cylindrical concrete specimens with a height of 50 mm and a diameter of 100 mm were made using plastic molds cut from a PVC tube with an inner diameter of 100 mm. Cracks were introduced into the specimens in an artificial way by introducing thin brass plates with a thickness of 300 µm into the fresh concrete upon casting [53]. The plates had a width of 60 mm and were positioned in the center of the cylindrical molds up to a depth of 25 mm. In order to incorporate the capsule based self-healing mechanism described in Section 2.2 for artificial cracks, the method described by Van Belleghem et al. [52] was used. Three holes with a diameter of 3.5 mm were drilled in the brass plates with an intermediate distance of 20 mm. The capsules, filled with a PU precursor, were put through these holes and glued on thin nylon threads, connected to the mold, to fix their position (Figure 1). The capsules were placed at 12.5 mm from the surface of the specimens. In this way, the position of the capsules was fixed at half the crack depth.

After the preparation of the molds, the concrete was cast and the specimens were stored in an air-conditioned room at a temperature of 20 °C and a relative humidity of more than 95% for 24 h. Subsequently, the specimens were demolded and stored in the same environment to cure until the age of 28 days. After the curing period, the plates were removed from all samples creating standardized cracks with a predefined width, depth and length of 0.3 mm, 25 mm and 60 mm, respectively. For the specimens with the self-healing properties the capsules broke due to the removal of the plates. In this way, the healing mechanism was triggered and the healing agent was released in the crack.

Since the exposure surface containing the artificially induced crack was a troweled surface with a certain roughness, it was mechanically flattened by cutting off a layer of approximately 1 mm. In this way, a perfectly flat test surface was obtained. This was also done for the uncracked specimens. For the specimens with self-healing properties, the flattening of the test surface occurred after the PU was given the time to harden completely for 48 h. All samples used for the accelerated chloride diffusion tests were also partially coated to ensure unidirectional chloride ingress. All different test series that were used in this research are listed in Table 2.

### 2.4. Natural Choride Diffusion Tests on Uncracked Concrete

To determine the proper mix specific input parameters for service life prediction of the uncracked concrete, the partially coated cylindrical specimens taken from cubes were stored in an aqueous 33 g/L NaCl solution at 20 °C after 28 days. This concentration corresponds with the normal Cl^−^ concentration in the North Sea. After 77, 139, 192, 262 and 311 days of exposure each time three cylinders were removed from the solution. Subsequently, 10 concrete powders were collected from each cylinder by grinding material in 2 mm layers parallel to the exposure surface. Determination of the total chloride concentration per ground layer of powder consisted of an acid-soluble extraction in a nitric acid solution followed by a potentiometric titration against silver nitrate cf. Mu [54].

### 2.5. Accelerated Choride Diffusion Tests on Uncracked, Cracked and Healed Concrete

**Figure 2:**
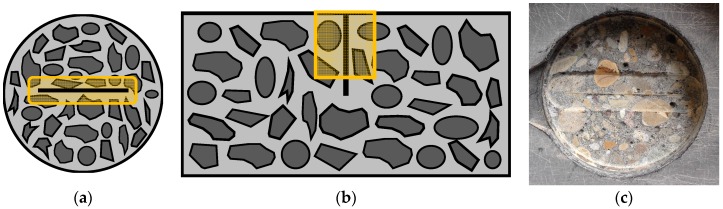
(**a**) Schematic top view of the 16 mm × 78 mm zone around the crack that was ground off; (**b**) schematic side view of the material ground away around the crack; and (**c**) top view of a specimen where a 2 mm thick layer of concrete was ground away around the crack.

In order to evaluate the efficiency of the proposed self-healing concrete against chloride ingress through cracks, accelerated chloride diffusion tests were done on the partially coated uncracked, cracked and healed specimens according to the standard NT Build 443, yet without pre-saturation of the samples in aqueous saturated Ca(OH)_2_ solution. An aqueous NaCl solution with a concentration of 165 g NaCl per dm^3^ solution was prepared for the accelerated test. All specimens were completely immersed in this solution and kept at a constant temperature of 20 °C. At exposure times of 49 and 133 days, three specimens of each test series were removed from the exposure solution. The chloride profiles of the specimens at the respective exposure times were determined by grinding off material in layers of 2 mm parallel to the exposed surface. A diamond coated drilling head with a diameter of 16 mm was used to grind away the material in a zone of 16 mm × 78 mm around the crack (Figure 2), similar to Maes et al. [33].

After the collection of the powders of all the ground concrete layers, the powders were dried in an oven at 105 °C for a minimum of 7 days. Subsequently, the total chloride content of each powder was determined in the same way as described in Section 2.4.

### 2.6. Chloride Profile Fitting

Under the assumption that the diffusion coefficient and surface chloride concentration remain constant in time, the chloride ingress can be estimated using the well-known formula suggested by Collepardi et al. [55] which is based on the second diffusion law of Fick (Equation (1)):

(1) $$C(x,t)=C_{0}+(C_{s}-C_{0})\cdot \left[1-\mathrm{erf}\left(\frac{x}{\sqrt{4\cdot D_{\mathrm{app}}\cdot t}}\right)\right]$$

with C(x, t), the chloride concentration at depth x and time t; C_0_, the initial chloride concentration (m%/binder); C_s_, the constant surface concentration (m%/binder); and erf(.), the error function and D_app_, the apparent diffusion coefficient (m^2^/s). It should be noted that D_app_ counts as a time-averaged value over the entire exposure period and not an instantaneous value. Values for C_s_ and D_app_ were estimated from Equation (1) by applying non-linear regression analysis on the experimental chloride profiles. The first layer (0–2 mm) of the chloride profiles was omitted in this regression analysis.

However, Visser et al. [56] showed that D_app_ does change with exposure time and therefore, this time dependency should be included in the prediction models. According to the same authors, the following updated formula should be in agreement with this requirement for the continuous exposure condition (Equation (2)):

(2) $$C(x,t)=C_{0}+(C_{s}-C_{0})\cdot \left[1-\mathrm{erf}\left(\frac{x}{\sqrt{4\cdot \frac{D_{0}}{\left(1-n\right)}{\left(\frac{t_{0}}{t}\right)}^{n}\cdot t}}\right)\right]C$$

with D_0_, the instantaneous diffusion coefficient (m^2^/s)) at reference time t_0_ which is usually 28 days (=0.0767 years) and n, the ageing exponent (−). In this equation there are three unknown variables (C_s_, D_0_ and n) that need to be optimized using the least-of-squares method. According to Visser et al. [56] it is important to fit all three variables simultaneously for all chloride profiles, measured at different exposure times. An initial estimation of the surface concentration and diffusion coefficient using Equation (1) followed by a subsequent fitting of the ageing exponent using an age function, will not result in optimum values for the three variables C_s_, D_0_ and n at once. Thus, also in this research the very same approach as proposed by Visser et al. [56] was adopted for the natural diffusion tests on uncracked concrete. The full dataset of all experimental chloride profiles obtained at the five considered exposure times (77, 139, 192, 262 and 311 days) with three replicates per exposure time was considered as a whole in order to estimate C_s_, D_0_ and n using Equation (2).

## 3. Service Life Prediction

The probabilistic limit state function (Equation (3)) used for estimating the time to chloride-induced steel depassivation looks almost identical to Equation (2) of Visser et al. [56].

(3) $$C_{\mathrm{crit}}=C_{0}+(C_{s}-C_{0})\cdot \left[1-\mathrm{erf}\left(\frac{d}{\sqrt{4\cdot k_{e}\cdot \frac{D_{0}}{1-n}\cdot {\left(\frac{t_{0}}{t}\right)}^{n}\cdot t}}\right)\right]$$

The only difference is that an environmental transfer variable k_e_ has been included to account for the fact that the temperature of the aqueous exposure solution during the laboratory diffusion tests differs from the actual seawater temperature in practice. In accordance with fib Bulletin 34 [57], variable k_e_ represents the typical Arrhenius function (Equation (4)).

(4) $$k_{e}=\exp\left(b_{e}\left(\frac{1}{T_{\mathrm{ref}}}-\frac{1}{T_{\mathrm{real}}}\right)\right).$$

Variable d (Equation (3)) corresponds with the concrete cover d on top of the reinforcing steel. Eurocode 2 prescribes a minimum concrete cover of 50 mm for construction class S6 which corresponds with a design service life of 100 years in exposure class XS2. Despite the fact that this concrete cover is strictly specified, the actual value is subject to variation due to the unavoidable small errors that may occur during the construction phase of a steel reinforced concrete element or structure. Therefore, it is a stochastic variable. As such, a standard deviation of 8 mm on the minimum cover was taken into consideration. The latter value is seen as appropriate for concrete without particular execution requirements [57]. The probabilistic distribution of the concrete cover was considered lognormal.

The critical chloride concentration, represented by C_crit_ in Equation (3), counts as the decisive criterion in the limit state function. Once the chloride concentration at the location of the steel rebar reaches this critical value, onset of active corrosion can start. In this case study, this event is seen as the end of service life. In [53] it has been experimentally verified in accordance with the method prescribed by RILEM TC 235 CTC—time-dependent monitoring of the corrosion potential of embedded reinforcing steel until onset of active corrosion—that this critical chloride concentration should be at least equal to 1.22 ± 0.02 m%/binder for the studied concrete composition under submerged exposure conditions. The same preliminary value has been adopted in this paper. As mentioned in [57], this variable follows a Beta distribution.

Reliability indices (β) and probabilities of failure (P_f_) as a function of time were calculated for Equation (3) using the First Order Reliability Method (FORM) available in the probabilistic Comrel software. Cf. fib Bulletin 34 [57], these parameters need to meet the requirements for the depassivation limit state (β ≥ 1.3 and P_f_ ≤ 0.10) to qualify for use in exposure class XS2. Table 3 summarizes the applied distributions and their characterizing parameters of all model input. The specific origin of all reported values for C_0_, C_s_ and D_0_ is discussed more in detail in Section 5.1 and Section 5.2.3. Note that the calculation for ordinary cracked concrete was done as follows. The model input of the uncracked concrete was used in combination with a concrete cover of only 25 mm instead of 50 mm to account for the presence of a 25 mm deep crack.

## 4. Life Cycle Assessment

In accordance with ISO 14040, the LCA consisted of four major steps: definition of goal and scope, inventory analysis, impact analysis and interpretation. The overall methodology followed is the same as the one that was adopted in by Van den Heede et al. [58].

### 4.1. Definition of Goal and Scope

The goal of this LCA was a quantification of the environmental impact reduction that could be achieved by using the proposed self-healing concrete instead of a more traditional 15% fly ash concrete in exposure class XS2. To do this in an adequate manner, the LCA study takes into account the difference in service life between traditional (cracked) concrete and the same concrete with self-healing properties. Therefore, a steel reinforced concrete slab with a design service life of 100 years and a variable load of 5 kN/m^2^ was chosen as functional unit (FU). As such, the extra material needed to repair the slab as soon as steel corrosion would be at risk was accounted for. For a slab repair, an extra concrete volume representing the 50 mm cover on top of the rebars plus the thickness of these rebars was taken into account. For the self-healing concrete slabs and its repairs the presence of one layer of 4150 capsules filled with PU precursor in the tensile zone of the slab was considered.

### 4.2. Inventory Analysis

For almost all concrete constituents, the life cycle inventory (LCI) data were collected from the renowned Ecoinvent database [59] (Table 4).

For the allocation of impacts related to the industrial by-product fly ash, the economic allocation coefficient as proposed by [41] was applied. This is 1.0% of the impact of the coal fired electricity production corresponding with the production of 1 kg fly ash. SP inventory data were compiled from an environmental declaration published by the EFCA [60]. The transport of each constituent to the concrete plant was not incorporated in the LCA since its environmental impact is always very case specific. The impacts associated with the production process at a ready-mix concrete plant were incorporated by the partial assignment of the following LCI from Ecoinvent: “Concrete, normal at plant/CH U”. It comprises the whole procedure of mixing 1 m^3^ of concrete, including all internal underlying sub-processes (transport, wastewater treatment, etc.).

As indicated in Table 4, the existing LCI for PU flexible foam in Ecoinvent was somewhat modified to make it more representative for the PU_HV and PU_LV that were used in this research. One important change for the PU_HV relates to the fact that the toluene diisocyanate (TDI) needed to be replaced with methylene diphenyl diisocyanate (MDI). Another distinct modification was the removal of the water for reaction with the PU precursor from the LCI, as this water is being provided by the moisture content of the concrete. The composition of the commercial PU_LV was not known. It could not be disclosed by its manufacturer for reasons of confidentiality. Consequently, a specific LCI with the correct values for the MDI, polyether polyol and inert hydrophobic compound content and its resulting emissions upon reaction could not be compiled for this healing agent. For now, the same composition as for the PU_HV was assumed for all LCA calculations until more information on the commercial healing agent PU_LV becomes available. The amount of PMMA sealant used to close the glass tubes was so small that this component could be omitted from the LCI.

### 4.3. Impact Analysis and Interpretation

CML-IA was used as impact method. It is an update of the CML 2 baseline 2000 method which was released by the Center of Environmental Science (CML) of Leiden University in 2013. It gives an eco-profile with ten baseline impact indicators regarding abiotic depletion (ADP, MJ fossil fuels), global warming (GWP, kg CO_2_ eq), ozone depletion (ODP, kg CFC-11 eq), human toxicity (HTP, kg 1,4-DB eq), freshwater aquatic ecotoxicity (FAETP, kg 1,4-DB eq), marine aquatic ecotoxicity (MAETP, kg 1,4-DB eq), terrestrial ecotoxicity (TETP, kg 1,4-DB eq), photochemical ozone creation (POCP, kg C_2_H_4_ eq), acidification (AP, kg SO_2_ eq) and eutrophication (EP, kg PO_4_ eq).

## 5. Results and Discussion

### 5.1. Natural Chloride Diffusion Tests on Uncracked Concrete

**Figure 3:**
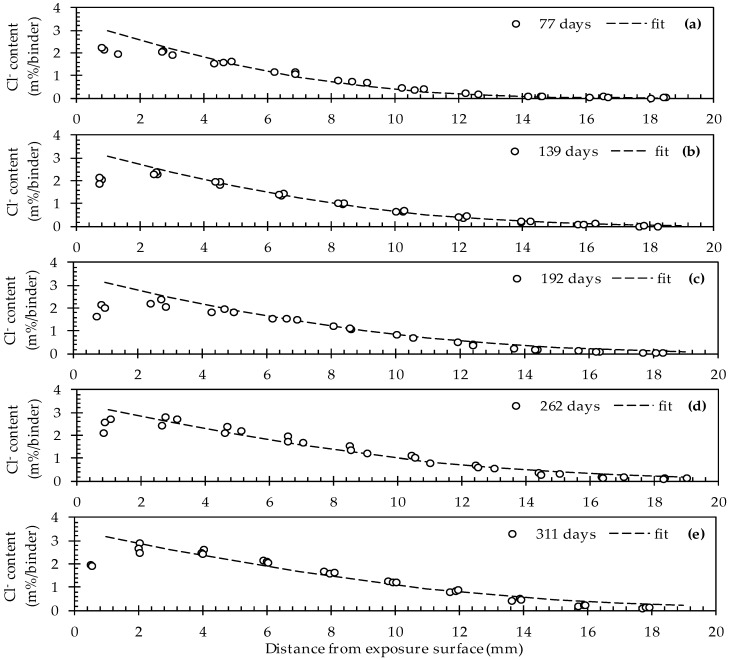
Experimental and fitted chloride profiles for uncracked concrete after (**a**) 77; (**b**) 139; (**c**) 192; (**d**) 262 and (**e**) 311 days of exposure in an aqueous 33 g/L NaCl solution.

The simultaneous fitting of C_s_, D_0_ and n cf. Visser et al. [56] resulted in theoretical chloride profiles that correspond rather well with the experimental data (Figure 3).

This seems to be the case for all five exposure times (77, 139, 192, 262 and 311 days). As such, the values for C_s_, D_0_ and n amount to 3.42 ± 0.05 m%/binder, 89 ± 3 mm^2^/years and 0.33 ± 0.04, respectively (Table 3). For this fit an *R*^2^ value of 0.978 was recorded. When looking at the evolution of the chloride profiles as a function of the exposure time it is clear that the chlorides move inwards. In other words, for a given distance from the exposure surface, the chloride concentration is gradually increasing. While for the first exposure time (77 d) the chloride profile reaches a stable value at a depth of around 14 mm. For the last exposure time (311 d) this event is only observed at a depth of around 18 mm onwards. Based on these results, it is quite evident that at a certain moment, the zone in direct contact with an embedded rebar will reach the critical chloride concentration that causes breakdown of the protective passivation layer on top of the rebar.

Note that the experimental data corresponding with the first ground layer (0–2 mm) of all five profiles clearly deviate from the fitted profiles. On the one hand, this is not surprising because the first layer was never considered in the fitting process (as mentioned in Section 2.6 and cf. NT Build 443). The reason for omitting the first layer relates to the fact that the chloride ingress close to the exposed surface is usually not diffusion controlled. It is part of the convection zone [57]. In addition, it is believed that increased calcium leaching in the first layer which is in close contact with the exposure solution tends to cause a decreased chloride binding capacity [61,62].

### 5.2. Accelerated Choride Diffusion Tests on Uncracked, Cracked and Healed Concrete

**Figure 4:**
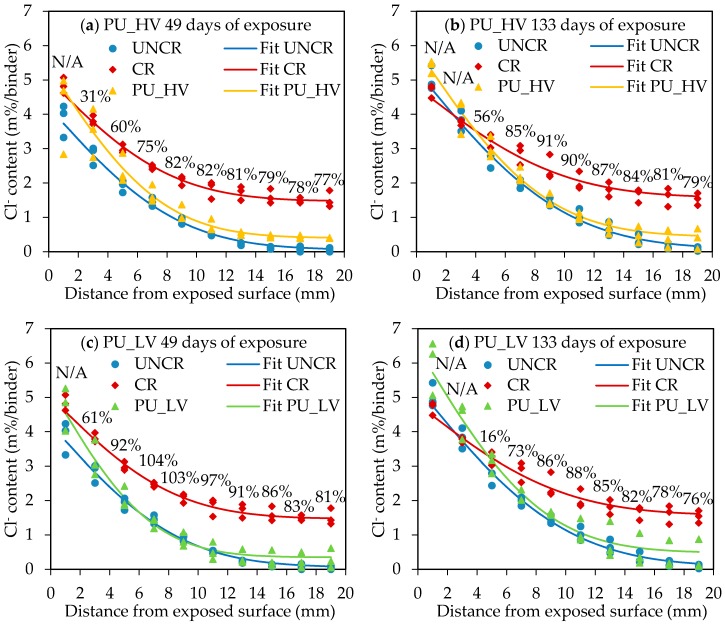
Experimental and fitted chloride profiles for (un)cracked and healed concrete with PU_HV after (**a**) 49 and (**b**) 133 days; and healed concrete with PU_LV after (**c**) 49 and (**d**) 133 days of exposure in 165 g/L NaCl solution. The self-healing efficiency (SHE) of each layer is denoted above the curves.

The experimentally obtained chloride profiles and corresponding profile fittings according to Equation (1) for all (un)cracked and healed specimens at the two exposure periods are shown in Figure 4.

#### 5.2.1. Influence of Cracks on Penetration of Chlorides

**Figure 5:**
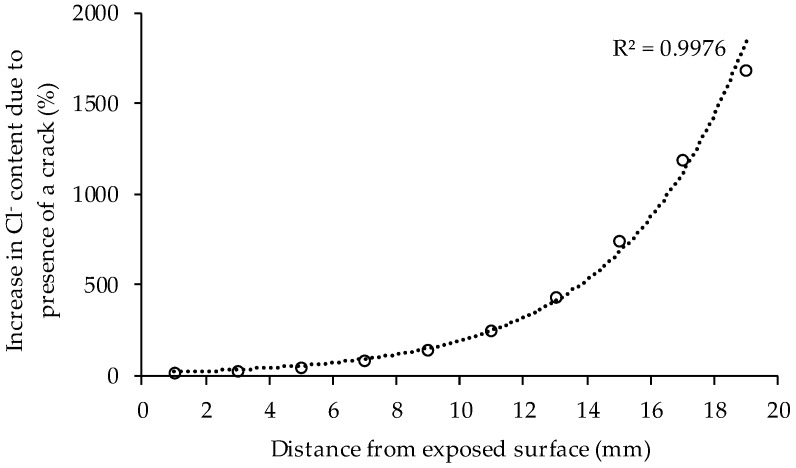
Increase of chloride concentration in function of the distance from the exposed surface due to the presence of a crack (exposure period of 49 days). The dotted line represents an exponential fit. The corresponding coefficient of determination (*R*^2^) is denoted in the graph.

The graphs in Figure 4 show clearly that cracks of 300 µm have a huge influence on the penetration of chlorides in concrete. After 49 days of exposure an increase of chloride content at every depth in the concrete is found due to the presence of the crack. The influence of the crack is rather limited close to the exposed surface, but increases nearly exponentially when going deeper in the concrete (Figure 5). Based on the fitted profiles in Figure 4, the chloride content at a depth of 18–20 mm was almost 18 times higher in cracked concrete compared to uncracked samples. The high increase in chloride content due to the presence of a crack from a depth below the surface of 10 mm onwards can be attributed mainly to the fact that the chloride ingress in cracked concrete in an early stage is dominated by the capillary absorption of the exposure solution in the crack.

At an exposure time of 133 days, the difference in chloride content between cracked and uncracked concrete was less pronounced than for 49 days of exposure. This is because the ingress of chlorides at later times was dominated by diffusion. Since there were already a lot of chlorides present in the concrete near the crack surface due to early capillary absorption, the diffusion of chlorides through the crack is slowed down compared to uncracked concrete. It was found that the maximum increase in chloride content between 49 and 133 days of exposure for the cracked concrete was 14%, while for uncracked concrete an increase of 27% (depth 0–2 mm) to 104% (depth of 14–20 mm) was found. However, the chloride content at an exposure time of 133 days at a depth of 18–20 mm was still 10 times higher due to the presence of a crack. Consequently, the presence of a 300 µm crack has a big influence on the chloride penetration in concrete, especially at larger depths below the exposed surface and thus possible locations of the steel reinforcement.

#### 5.2.2. Evaluation of Crack Healing

Figure 4a,b gives the chloride profiles and fittings for the specimens healed with the high viscosity polyurethane (PU_HV) at 49 and 133 days of exposure, respectively. Figure 4c,d shows the chloride profiles and fittings for the specimens healed with the low viscosity polyurethane (PU_LV) at 49 and 133 days of exposure, respectively. The fitted chloride profiles of the healed specimens are almost completely in between the profile of the uncracked and the cracked specimens, except for the first layer (0–2 mm) in the case of 49 days of exposure and the first two layers (0–4 mm) in the case of 133 days of exposure. A possible reason for the high chloride content in the first layers of the specimens with a healed crack could be the fact that the crack healing was not perfect. At the surface of the healed cracks it could be seen that some very small parts of the crack were not well sealed. It is thus possible that the exposure solution entered into the top part of the crack through these small imperfections in the crack healing. The imperfections might be caused due to the mechanical removal of the top layer of the specimens to obtain a flat test surface. However, deeper inside the crack (and thus closer to the capsules) there might be a more complete crack filling which prevents the chlorides from penetrating deeper into the crack. As a result, there is an accumulation of chlorides in the top layers of the specimens with a healed crack. However, the main deterioration mechanism caused by the ingress of chlorides is chloride induced reinforcement corrosion. Since the reinforcement in a concrete element is usually located at a depth much higher than 4 mm, a high chloride concentration in the top layers (0–4 mm) does not directly have a negative impact on the durability of the element.

When going from the top layers deeper inside the concrete, the chloride concentration drops very fast for the healed specimens. This can clearly be seen from the steep slopes of the chloride profiles of the healed specimens at depths from 0 to 6 mm below the exposed surface. From a depth of 6 mm onwards, healing of cracks decreases the chloride content to a large extent compared to unhealed cracks. The hardened polyurethane inside the cracks clearly forms a partial barrier against immediate ingress of chlorides through the cracks. In order to make clear conclusions about the performance of the self-healing mechanism, a self-healing efficiency at every depth i below the exposed surface (SHE_i_) was defined according to Van den Heede et al. [53] (Equation (5)):

(5) $$SHE_{i}=\left(\frac{Cl_{\mathrm{CR},i}^{-}-Cl_{\mathrm{HEAL},i}^{-}}{Cl_{\mathrm{CR},i}^{-}-Cl_{\mathrm{UNCR},i}^{-}}\right)\cdot 100$$

with $Cl_{\mathrm{CR},i}^{-}$, the chloride content of the cracked specimen at depth i (m%/binder); $Cl_{\mathrm{HEAL},i}^{-}$, the chloride content of the healed specimen at depth i (m%/binder); and $Cl_{\mathrm{UNCR},i}^{-}$, the chloride content of the uncracked specimen at depth i (m%/binder). All chloride contents were based on the values of the fitted curves.

As mentioned before, the fitted profile of the healed specimens is located above the fitted profile for the cracked specimens in the first or first two layers for an exposure time of 49 or 133 days, respectively. According to the definition of the self-healing efficiency (Equation (5)) this would mean that there is a negative SHE in these first layers. This was denoted as not applicable (N/A). For all other layers in the profile, the SHE (%) is given above the curves in Figure 4. Generally the SHE is first increasing with depth and reaches a maximum in the layers at 8 to 12 mm below the exposed surface. The maximum healing performance in these layers is a result of the capsule location. The capsules were placed in the molds on nylon threads at a position of 12.5 mm from the exposed surface (see Figure 1). After removal of the outermost 1 mm layer of the specimens to obtain a flat test surface, the location of the capsules is approximately 11.5 mm below the exposed surface. From 12 to 20 mm depth there is a slight decrease of SHE. However, from a depth of 6 mm onwards, the SHE was always 75% or higher for the two types of polyurethane and at the different exposure periods. This big reduction of chloride concentration in a crack due to autonomous self-healing will have important benefits for the durability of reinforced concrete since a much lower amount of chlorides will reach the steel reinforcement through the cracks. The time to reach the critical chloride concentration in a marine environment at the location of the reinforcing steel will thus be much higher in the case of self-healing concrete. This will cause an extension of the service life of the concrete elements (see Section 5.3).

The SHE gives an idea about the general performance of the self-healing mechanism regarding the durability in chloride environments. However, it might be interesting to have a look at the individual results as well. In the case of crack healing with the high viscosity polyurethane (PU_HV) at an exposure time of 49 days, there is not much variability between the different healed specimens, especially not in the layers from 12 to 20 mm. All healed specimens showed more or less the same healing performance, but none of them behaved as uncracked concrete. At 133 days of exposure, the mean healing efficiency of PU_HV was even higher than at 49 days, but the variability between the different specimens was also higher. One of the healed specimens behaved as almost uncracked with a SHE at the deepest ground layer (18–20 mm) of 97%. For the other two specimens in this series, the SHE at this depth was 74% and 58%.

For crack healing with the low viscosity polyurethane (PU_LV) similar results were found at both exposure times. Two out of three specimens were almost perfectly healed, behaving very similar to uncracked concrete. One specimen however performed much worse than the others. This can be seen clearly in Figure 4c,d as the chloride contents for one specimen at depths larger than 10 mm are located well above the fitted line. At an exposure time of 49 days the SHE at a depth of 18–20 mm for the three individual specimens was 91%, 102% and 65%. At 133 days of exposure the SHE value at a depth of 18–20 mm of the two almost perfectly healed specimens was 95% and 96%, whereas a value of only 42% was found for the third specimen. From Figure 4c,d it can be seen that the healing performance of the worse performing specimens is especially bad from a depth of 10 mm onwards, thus in the layers below the capsules. An explanation for the healing behavior of both polyurethane types can be found in previous research by Van den Heede et al. [32]. In that study, healing of artificial cracks by both types of polyurethane (PU_HV and PU_LV) was evaluated in mortar prisms by splitting the samples and analyzing the spread region of the polyurethanes on the crack faces. It was found that the high viscosity polyurethane was able to cover 79% to 92% of the crack face, while a coverage of only 13% to 21% was found for the cracks healed with the low viscosity polyurethane. Moreover, for PU_LV crack filling was only observed at the bottom of the crack (close to the test surface). PU_HV is thus able to fill up a large amount of the crack, but always has some small imperfections in the healing. Consequently, this healing agent is able to heal cracks partially and reduce the chloride ingress through the cracks by approximately 75%, but it almost never reaches a behavior similar to uncracked concrete. Crack healing with PU_LV causes only a smaller part of the crack to be healed between the location of the capsules and the exposed surface. The part of the crack below the capsules may remain unhealed. Nevertheless, if the crack healing at the outer part is perfect, it will block the ingress of chlorides through the crack completely. When the crack healing is not perfect however, chlorides may enter the crack and reach the unhealed part below the capsules. According to the authors, this is a reason why one out of three specimens healed with PU_LV performed worse than the others.

#### 5.2.3. Surface Chloride Concentration and Apparent Diffusion Coefficient

From the profile fitting of the experimental chloride profiles, the values for C_s_ and D_app_ were determined using Equation (1). However, the model for chloride ingress based on Fick’s second law of diffusion (Equation (1)) was developed based on sound, uncracked concrete. C_0_ in Equation (1) is the initial chloride concentration in the concrete, which reaches a stable value far enough away from the surface exposed to chlorides. In the case of cracked (or healed) concrete, the experimental chloride profiles in the cracked zones also have a tendency towards a stable value at a depth larger than 14 mm below the exposed surface (see Figure 4), but this value is obviously not the initial chloride content of the concrete. In order to use Equation (1) for fitting the experimental chloride profiles of the cracked and healed specimens, C_0_ was assumed to be the chloride content of the layer at 18–20 mm depth from the exposed surface.

Table 5 and Table 6 give an overview of the estimated values of C_s_ and D_app_ from Equation (1) respectively with their standard deviation σ. The surface chloride concentration of all series, except CR, increased due to a longer exposure time. This is logical, since there is more ingress of chlorides with time. For the specimens with healed cracks larger values of C_s_ were found than for (un)cracked samples. The reason for the high surface chloride content in the crack zone of the healed samples is already explained in Section 5.2.2.

From Table 6 it can be seen that the apparent diffusion coefficient for all test series is decreasing for a longer exposure time. This is in agreement to what is found in the literature [56,63,64,65,66]. At 133 days of exposure, D_app_ is always approximately reduced by half compared to 49 days of exposure. The presence of a (healed) crack thus has a limited influence on the development of the diffusion coefficient over time. At both exposure times, D_app_ is largest for uncracked concrete and has the smallest value for healed concrete (especially PU_LV). The fact that the diffusion coefficient of cracked concrete is lower than the one of uncracked concrete is mainly because chlorides do not only enter the concrete through diffusion from the exposed surface. The chloride solution can easily enter into the crack and there is also ingress of chlorides perpendicular to the crack faces. This causes the concentration gradient of chlorides in the concrete to be different and will consequently have an effect on the apparent diffusion coefficient. In the case of self-healing concrete there is much less immediate ingress of chlorides deep into the crack, since this is (partly) blocked by the hardened polyurethane inside the crack. Therefore, the fitted curves of the healed specimens look similar to the ones for uncracked specimens (see Figure 4). The main difference is that the healed specimens have a higher C_s_ and C_0_. Due to the increase in C_s_, the fitted curve for the healed specimens is much steeper than for the uncracked specimens. Consequently, the apparent chloride diffusion coefficient is lower (Table 6).

For a service life prediction of self-healing concrete, input of the surface chloride concentration and diffusion coefficient is required. However, the values of D_app_ and C_s_ found by the accelerated chloride diffusion test could not be used directly since the exposure solution used for this test is not realistic. Instead, an estimation of the diffusion coefficient and the surface chloride concentration of healed concrete (PU_HV and PU_LV) was made. First, the ratio of D_app_ (or C_s_) for healed concrete and uncracked concrete was calculated. This ratio was then multiplied by the realistic value of the instantaneous diffusion coefficient (and surface chloride concentration) of uncracked concrete found with the natural diffusion test (see Section 5.1). These values were then used as input for the service life prediction model (Table 3: C_s__PU_HV_49 d/133 d, C_s__PU_LV_49 d/133 d, D_0__PU_HV_49 d/133 d, D_0__PU_LV_49 d/133 d). The same was done to make an estimation of C_0_ for self-healing concrete as input for the service life prediction model (Table 3: C_0__PU_HV_49 d/133 d, C_0__PU_LV_49 d/133 d). For ordinary cracked concrete, these ratios did not need to be calculated since that service life prediction was simply done by assuming all model input for the uncracked concrete in combination with a reduced concrete cover of only 25 mm to account for the presence of the crack.

### 5.3. Service Life Prediction

Given the fact that a 100% self-healing efficiency could not be achieved with either of the two encapsulated PU-based healing agents, the expected time to chloride-induced steel depassivation of the PU_HV and PU_LV concrete is less than the 97-year timespan that was found for the ideal uncracked condition of the concrete cover on top of the reinforcement. Nevertheless, the service life prediction outcome of 60 years (PU_HV) and 94 years (PU_LV) resulting from the accelerated diffusion tests with a 49 d exposure period is still a lot more than the seven years that was calculated for the cracked concrete without presence of an embedded healing mechanism (Table 7). The same holds true when the service life prediction results (PU_HV: 82 years, PU_LV: 76 years) based on the accelerated diffusion tests with a 133 d exposure period are taken into consideration. Depending on the exposure period (49 d vs. 133 d), both the concrete with encapsulated PU_HV and PU_LV have a better service life performance. Most probably the number of chloride diffusion test samples to determine the conversion factors to go from C_0_UNCR_, C_s_UNCR_, and D_0_UNCR_ to C_0_PU_HV/LV_, C_s_PU_HV/LV_, and D_0_PU_HV/LV_ per exposure time (see Table 3) was too small to account for all possible variation per type of healing agent. Consequently, based on the results that are currently available, no real preference for either of the two healing agents could be put forward. It would evidently be recommended to use more samples in the future to sort this out with more certainty.

With a design service life set at 100 years, steel reinforced concrete elements, like for instance the slab that was considered in this research, would need to be repaired only once in course of the predefined timespan when using either one of the two studied self-healing concrete types (Table 7). When using ordinary (cracked) concrete, repair works (i.e., replacement of the 50 mm concrete cover on top of the rebars) would need to be done on a much more regular basis (14 repairs). It should be noted that both self-healing concrete types seem to have a very similar performance in terms of repair frequency. Moreover, the repair frequency is the same as for the ideally preferred uncracked state of the concrete. Obviously, this depends on the chosen design service life of the concrete. The now observed similarity in repair frequency life is only valid for a design service life of 100 years.

### 5.4. Life Cycle Assessment

**Figure 6:**
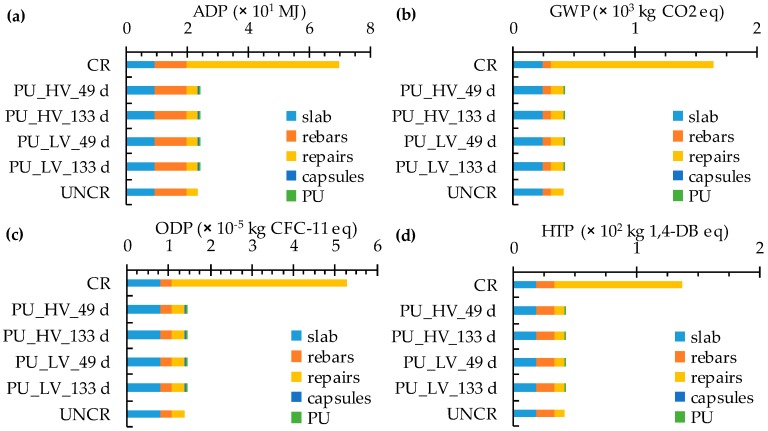

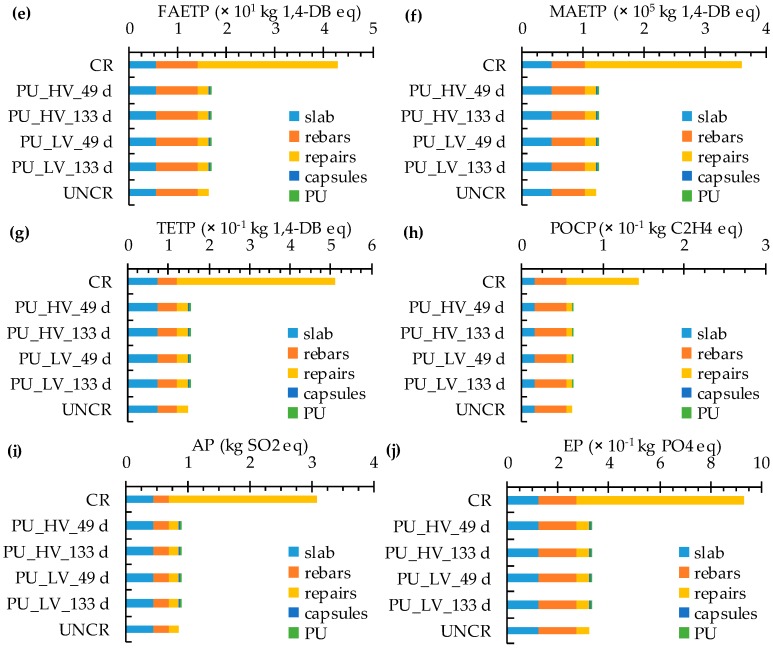
The ten CML-IA baseline impact category indicators for steel reinforced concrete slabs (variable load: 5 kN/m^2^, design service life: 100 years, including repairs) made with (un)cracked and self-healing concrete with encapsulated PU_HV and PU_LV: (**a**) ADP; (**b**) GWP; (**c**) ODP; (**d**) HTP; (**e**) FAETP; (**f**) MAETP; (**g**) TETP; (**h**) POCP; (**i**) AP; and (**j**) EP.

When assessing the ten CML-IA baseline impact indicators for the considered functional unit (i.e., steel reinforced concrete slab with a given load and service life in exposure class XS2), a high sustainability potential seems to be present for the self-healing concrete with encapsulated PU precursor in comparison with ordinary (cracked) concrete (Figure 6). ADP (−66%), GWP (−74%), ODP (−73%), HTP (−69%), FAETP (−61%), MAETP (−65%), TETP (−70%), POCP (−56%), AP (−71%) and EP (−64%) values that have been recorded for the self-healing concrete are all substantially lower. This can mainly be explained by the fact that the regular time dependent replacement of the cover for the cracked concrete imposes a huge environmental burden onto the slab. On the other hand, the presence of the encapsulated PU precursor to reduce the repair frequency to only one time has a negligible impact on all ten baseline impact categories (1%–4% of the impact of one steel reinforced slab including one repair). The encapsulated PU precursor was found to contribute the most (3%–4%) to the HTP, FAETP, TETP, POCP, AP and EP.

Some critical remarks should be made regarding the very positive LCA calculation outcome for the self-healing concrete. First of all, the difference in impact between the two types of healing agents could not be assessed because the exact composition of the commercial PU_LV precursor was not known. Nevertheless, given the minimal contribution of the PU precursor to the overall impact and the fact that, apart from the presence of inert, hydrophobic compounds, the main components of the commercial PU_LV are the same as for the in-house developed PU_HV, no huge differences in impact between the two types of steel reinforced self-healing concrete slabs are expected. Evidently, further in-depth investigation on the most likely composition of the PU_LV is for the moment still ongoing to find further confirmation for this.

Secondly, the environmental scores obtained now are only valid for a repair scenario that consists of replacing the entire 50 mm concrete cover until just after the position of the rebars. As an alternative, a repair strategy consisting of a manual injection with the same healing agents could be considered. However, in order to obtain a reliable evaluation for that option one should subject the same concrete with 300 µm wide cracks that have been healed manually to the same accelerated diffusion tests to quantify the difference in repair efficiency and service life performance with the self-healing concrete. These data are not yet available for the moment.

Moreover, when making the comparison between manually healed and self-healing concrete, the functional unit for LCA should be redefined. In terms of material use the impacts of the slabs should be more or less equal when assuming a more or less equal healing efficiency for manual repair and autonomous self-healing. Only the impacts related to the presence of the capsules are missing in the case of the former strategy.

However, this approach does not take into account that manual injection requires the use of specific equipment with possible additional environmental effects. In addition, the structure needs to be accessible to allow for repair. In other words, the fact that often huge impacts—related to traffic jams when dealing with road structures, temporal change in production capacity when dealing with industrial infrastructure or temporal function loss when dealing with concrete buildings—can be avoided by going for the autonomous healing strategy. In fact, this aspect is also of relevance when evaluating the impacts associated with concrete cover replacement as repair strategy. It would impose even higher impacts to the cracked reference concrete which would increase the sustainability potential of the self-healing concrete even more. All in all, it would certainly be worthwhile to investigate other possible functional units that account for these indirect structure accessibility related environmental impacts in future research as well.

## 6. Conclusions

The accelerated chloride diffusion test on uncracked and cracked concrete showed the big influence of a 300 µm wide crack on the chloride ingress in concrete. Close to the exposed surface, the influence was rather limited, but increased nearly exponentially in depth. The chloride content at the deepest measured layer (18–20 mm) was 18 times higher due to the presence of a crack at 49 days of exposure and 10 times higher at 133 days of exposure.

In the case of concrete where the crack was healed by encapsulated polyurethane, the accelerated chloride diffusion tests showed that the hardened polyurethane inside the cracks formed a partial barrier against immediate ingress of chlorides through the cracks. Perfect healing of the crack, so that the concrete would behave as uncracked, was never possible. However, from a depth of 6 mm onwards, the self-healing efficiency for both types of polyurethane at the two tested exposure times was always 75% or higher. This reduction of chloride concentration in the zone around the crack will have important benefits for the durability of reinforced concrete since a much lower amount of chlorides will reach the steel reinforcement through the cracks.

The autonomous healing technique used for this research was manual and of course it should still be extended to a real healing technique for construction practice. In previous research, it was already proven that the proposed healing mechanism works to heal realistic cracks [20,34]. For real construction elements there are basically two ways to provide the capsules with healing agent in the concrete matrix. A first way would be prefabrication of a whole net of capsules that can be placed inside the mold of an element. In this way the capsules can also be provided specifically in the regions where crack formation is feared. A second way is adding the capsules to the concrete mix. Ongoing research is performed in the CAPDESIGN project on the development of capsules that can survive the mixing process.

Probabilistic service life prediction to estimate the time to chloride-induced steel depassivation showed that steel-reinforced self-healing concrete slabs containing one layer of 4150 PU-filled capsules in the tensile zone would only require repair works (i.e., replacement of the concrete cover) after 60–94 years. For ordinary cracked concrete slabs, repair would already be needed after seven years. In other words, a substantial service life extension seems indeed possible by embedding encapsulated PU precursor as autonomous healing mechanism in concrete. Based on the currently available prediction outcome, no preference could be formulated for the high or low viscosity PU precursor as encapsulated healing agent. It should also be noted that the now obtained service life information still needs to be verified and fine-tuned with more accurate numerical modeling of the chloride ingress in (un)cracked and self-healing concrete. This specific aspect is at this moment still under investigation and will be dealt with in a next stage of the ISHECO project.

Life cycle assessment calculations in SimaPro 8 for the same steel reinforced concrete slabs with inclusion of the required number of repairs with a 100-year lifespan—the functional unit of this LCA—mark substantial reductions in environmental impact for all ten CML-IA baseline impact indicators (56%–75%) when self-healing concrete would be used instead of ordinary (cracked) concrete. The difference in repair frequency (1 vs. 14) mainly explains this. The overall impact of the presence of encapsulated PU precursor turned out to be negligible.

## Figures and Tables

**Table 1:** Mix proportions and properties of the concrete mixture.

**Concrete Composition**	
Sand 0/4 (kg/m^3^)	696
Aggregates 2/8 (kg/m^3^)	502
Aggregates 8/16 (kg/m^3^)	654
CEM I 52.5 N (kg/m^3^)	317.6
Fly Ash (kg/m^3^)	56
Water (kg/m^3^)	153
Superplasticizer (mL/kg binder)	3.0
**Mix Properties**	
W/B (−)	0.41
FA/B (%)	15
Slump class	S3
Strength class	C40/50

**Table 2:** Specimen test series (*n* = number of specimens).

Code	Description of the Test Series	*n*
UNCR	Uncracked concrete specimens	6 ^a^ + 15 ^b^
CR	Concrete specimens containing a standard crack	6 ^a^
PU_HV	Concrete specimens containing a standard crack autonomously healed by the high viscosity polyurethane	6 ^a^
PU_LV	Concrete specimens containing a standard crack autonomously healed by the low viscosity polyurethane	6 ^a^

^a^ Specimens used for accelerated chloride diffusion test; ^b^ Specimens used for natural chloride diffusion test.

**Table 3:** Input to the probabilistic diffusion test based limit state function for estimating the time to chloride-induced steel depassivation for (un)cracked and healed concrete.

Variable	Distribution	Mean	Stdv.	Upper Limit	Lower Limit
C_0_UNCR_ (m%/binder)	Normal	0.06 ^a^	0.01	–	–
C_0_PU_HV_49 d_ (m%/binder)	Normal	0.41 ^b^ (=6.80 × 0.06)	0.01	–	–
C_0_PU_HV_133 d_ (m%/binder)	Normal	0.34 ^b^ (=5.71 × 0.06)	0.01	–	–
C_0_PU_LV_49 d_ (m%/binder)	Normal	0.36 ^b^ (=5.93 × 0.06)	0.01	–	–
C_0_PU_LV_133 d_ (m%/binder)	Normal	0.38 ^b^ (=6.32 × 0.06)	0.01	–	–
d (mm)	Lognormal	50/25	8	–	–
b_e_ (K)	Normal	4800	700	–	–
T_ref_ (K)	Constant	293	–	–	–
T_real_ (K)	Normal	283	5	–	–
t_0_ (years)	Constant	0.0767 (28 d)	–	–	–
C_s_UNCR_ (m%/binder)	Normal	3.42 ^a^	0.05	–	–
C_s_PU_HV_49 d_ (m%/binder)	Normal	4.35 ^b^ (=1.27 × 3.42)	0.05	–	–
C_s_PU_HV_133 d_ (m%/binder)	Normal	3.84 ^b^ (=1.12 × 3.42)	0.05	–	–
C_s_PU_LV_49 d_ (m%/binder)	Normal	4.27 ^b^ (=1.25 × 3.42)	0.05	–	–
C_s_PU_LV_133 d_ (m%/binder)	Normal	4.17 ^b^ (=1.22 × 3.42)	0.05	–	–
D_0_UNCR_ (mm^2^/years)	Normal	89 ^a^	3	–	–
D_0_PU_HV_49 d_ (mm^2^/years)	Normal	69 ^b^ (=0.77 × 89)	3	–	–
D_0_PU_HV_133 d_ (mm^2^/years)	Normal	68 ^b^ (=0.76 × 89)	3	–	–
D_0_PU_LV_49 d_ (mm^2^/years)	Normal	54 ^b^ (=0.61 × 89)	3	–	–
D_0_PU_LV_133 d_ (mm^2^/years)	Normal	63 ^b^ (=0.71 × 89)	3	–	–
n (−)	Beta	0.33	0.04	0.00	1.00
C_crit_ (m%/binder)	Beta	1.22	0.02	0.00	2.00

^a^ see Section 5.1; ^b^ see Section 5.2.3.

**Table 4:** Overview of the Ecoinvent life cycle inventory (LCI) data used.

Constituent	LCI Description Ecoinvent
Sand	Sand, at mine/CH U
Gravel 2/8 and 8/16	Gravel, round, at mine/CH U
CEM I 52.5 N	Portland cement, strength class Z 52.5, at plant/CH U
Fly ash	partially contains: ‘Electricity, hard coal, at power plant/BE U’, through economic allocation
Water	Tap water, at user/CH U
Glass capsule	Glass tube, borosilicate, at plant/DE U
PU-based healing agent	Polyurethane, flexible foam, at plant/RER U (modified)

**Table 5:** Estimated values of C_s_ for the different test series at exposure times of 49 and 133 days.

Specimen Series	49 Days		133 Days	
	C_s_ (m%/Binder)	σ (m%/Binder)	C_s_ (m%/Binder)	σ (m%/Binder)
UNCR	4.210	0.137	5.272	0.163
CR	5.007	0.177	4.795	0.206
PU_HV	5.355	0.334	5.917	0.273
PU_LV	5.258	0.379	6.429	0.394

**Table 6:** Estimated values of D_app_ for the different test series at both exposure times.

Specimen Series	49 Days		133 Days	
	D_app_ (m^2^/s) × 10^−12^	σ (m^2^/s) × 10^−12^	D_app_ (m^2^/s) × 10^−12^	σ (m^2^/s) × 10^−12^
UNCR	5.634	0.341	2.704	0.163
CR	5.161	0.467	2.597	0.316
PU_HV	4.347	0.512	2.062	0.189
PU_LV	3.442	0.441	1.920	0.230

**Table 7:** Service life prediction and repair frequency for cracked (CR), self-healing (SH) and uncracked (UNCR) concrete in marine environments (exposure class XS2).

Concrete	CR	PU_HV	PU_LV	UNCR
Service life (years)	7	60 (49 d)/82 (133 d)	94 (49 d)/76 (133 d)	97
# repairs	14×	1 × (49 d)/1 × (133 d)	1 × (49 d)/1 × (133 d)	1×
